# Methotrexate and Cyclosporine Treatments Modify the Activities of Dipeptidyl Peptidase IV and Prolyl Oligopeptidase in Murine Macrophages

**DOI:** 10.1155/2008/794050

**Published:** 2008-02-25

**Authors:** R. A. Olivo, N. G. Nascimento, C. F. P. Teixeira, P. F. Silveira

**Affiliations:** Laboratory of Pharmacology, Butantan Institute, 05503-900 São Paulo, Brazil

## Abstract

Analysis of the effects of cyclosporine A (25–28 mgkg^−1^) and/or methotrexate (0.1 mgkg^−1^) treatments on dipeptidyl peptidase IV (DPPIV) and prolyl oligopeptidase (POP) activities and on algesic response in two distinct status of murine macrophages (M*φ*s) was undertaken. In resident M*φ*s, DPPIV and POP were affected by neither individual nor combined treatments. In thioglycolate-elicited M*φ*s, methotrexate increased DPPIV (99–110%) and POP (60%), while cyclosporine inhibited POP (21%). Combined treatment with both drugs promoted a rise (51–84%) of both enzyme activities. Only cyclosporine decreased (42%) the tolerance to algesic stimulus. Methotrexate was revealed to exert prevalent action over that of cyclosporine on proinflammatory M*φ* POP. The opposite effects of methotrexate and cyclosporine on POP activity might influence the availability of the nociceptive mediators bradykinin and substance P in proinflammatory M*φ*s. The exacerbated response to thermally induced algesia observed in cyclosporine-treated animals could be related to upregulation of those mediators.

## 1. INTRODUCTION

Macrophage (M*ϕ*) is considered the main effector cell type of the immune system. Under stimulation, this cell is activated by a process involving morphological, biochemical, and functional changes [1]. Among the relevant enzyme activities on M*ϕ* functions are the membrane-bound (M) and soluble (S) dipeptidyl peptidase IV (DPP IV) and the S prolyl oligopeptidase (POP) [2]. DPPIV cleaves substance P [3] and inflammatory mediators such as interferon-gamma (IFN*γ*), chemokines and proinflammatory cytokines [4], while POP cleaves the nociceptive mediators bradykinin and substance P [5]. During inflammation, nonneuronal cells such as M*ϕ* produce a variety of chemical mediators that can act on nociceptive neurons [6]. On the other hand, nociceptive mediators such as bradykinin [7, 8] and substance P [9, 10] act on the immune response and M*ϕ* functions.

Methotrexate (MTX) and cyclosporine (CsA) are immunomodulators that belong to the most commonly used group of drugs for cytotoxic therapy. However, their exact mechanisms of action are not yet clarified. Although MTX and CsA have been used alone [11] or in a combined therapy [12] for inflammatory and painful chronic disease treatments, experimental and clinical studies are needed to determine the extent to which MTX and CsA treatments affect the M*ϕ* functions and, more specifically, its peptidases with hydrolytic ability on inflammatory and nociceptive mediators. It is known that M*ϕ*s functions are unchanged or reduced in the presence of CsA. The reduction includes in vitro interleukin-1 generation [13], chemotaxis [14], prostaglandin E_2_ production [15], procoagulant activity [16], and major histocompatibility complex (MHC) [17] and inducible nitric oxide synthase [18] expressions. CsA also reduces phorbol 12-myristate 13-acetate-dependent superoxide anion and H_2_O_2_ production in vitro by resident (RE) murine M*ϕ*s, which are abolished when M*ϕ*s are in the proinflammatory state [19]. MTX, but not CsA, is able to enhance in vitro spreading of murine peritoneal M*ϕ*s [20]. MTX is also known to inhibit production of cytokines induced by T-cell activation. Interleukin (IL)-4, IL-13, IFN gamma, tumor necrosis factor-alpha (TNF*α*) and granulocyte-macrophage colony-stimulating factor are among the cytokines inhibited by MTX [21].

The ex vivo isolated RE and thioglycollate broth medium-elicited (TGE) M*ϕ* models mimic, respectively, the in vivo basal and proinflammatory status of this cell. The proinflammatory M*ϕ*s are present in acute and chronic inflammation as major players in the generation and release of a variety of inflammatory and nociceptive mediators. A possible relationship between the ex vivo TGE M*ϕ* DPPIV and POP activity levels with the in vivo excitability to thermal pain stimulus could highlight the in vivo role of these peptidases through their actions on those inflammatory and nociceptive mediators after their generation and release by M*ϕ* s in inflammatory and painful diseases. This study aims to analyze the interference of MTX and CsA, each one daily administered alone or combined during 21 days, on the activity levels of S DPPIV and POP, and MDPPIV in two distinct status of ex vivo isolated peritoneal murine M*ϕ*s—the noninflammatory RE and the proinflammatory TGE cells—as well as whether the excitability to thermal pain stimulus could be altered by these drugs or correlated to M*ϕ* status and peptidase activities.

## 2. MATERIALS AND METHODS

### 2.1. Animals and treatments

Healthy Swiss strain mice, males, weighing 18–20 g, were maintained in a restricted-access room with controlled temperature of 25°C, relative humidity of 65.3 ± 0.9%, and 12 h light:12 h dark photoperiod (lights on at 6:00 am), and were housed in cages (inside length × width × height of 56 × 35 × 19 cm) with a maximum of 20 mice per cage, with food and tap water ad libitum.

Animals were subcutaneously (s.c.) injected, once a day, with 50 *μ*L of cyclosporine A (CsA) (10 mg CsA/mL ricine oil (starting dose: 25–28 mg/kg BW) or of ricine oil (control), during 21 days. Other groups were administered by gavage (p.o.) of 0.2 mL, once a day, with methotrexate (MTX) dissolved in saline 0.9% (starting dose: 0.1 mg/kg BW) or with saline (control), during 21 days. A fourth group and its corresponding control were simultaneously submitted to treatments with both drugs and both vehicles, respectively, in the same scheme as described above. Subsequently, M*ϕ*s were collected from individuals of each group (treated and controls of MTX and/or CsA). The regimen of treatment with MTX [22] and/or CsA [23] used in this study was chosen by its well-known immunosuppressive effect.

The animal and research protocols used in this study are in agreement with the Brazilian Council Directive (COBEA-BRAZIL) and were approved by the Ethics Committee of the Instituto Butantan.

### 2.2. Hot-plate nociceptive test

This test was employed based on the method of Jacob and Ramabadran [24]. Mice were placed on a metal surface kept at 64.5°C ± 0.5°C. Results are expressed as the latency time to the observed licking of both anterior feet (latency of response).

### 2.3. Obtention of RE and TGE macrophages

The peritoneal lavage was performed in half of each group (treated and controls of MTX and/or CsA) after a gentle massage of the abdominal wall. Then, the peritoneal fluid, containing M*ϕ*s, was collected. Aliquots of the washes were used to determine the total number of peritoneal cells in a Neubauer’s chamber after dilution (1:20, v/v) in Turk solution (0.002 g gentian violet in 500 mL 3% acetic acid). The predominance of mononuclear cells in the washes was confirmed by light microscopic analysis of smears stained with Hema^3^. The cell population consisted in proximally 99% of M*ϕ*s, as determined by morphological criteria. Washes were then centrifuged at 200 X*g*, 6 minutes, 22°C, and the pellet obtained resuspended in 2.0 mL of 10 mM Tris-HCl, pH 7.4.

The other half of each group was also submitted to peritoneal lavage, which was performed, according to the above description, 4 days after IP administration of 1.0 mL of 3% thioglycollate broth medium (TG). The cell population in the washes of these TG-treated mice consisted of more than 95% of M*ϕ*s, as determined by morphological criteria.

The number of obtained M*ϕ*s from peritoneal lavage was about 4 × 10^6^/mL in TG-treated (TGE-M*ϕ*s) and about 1 × 10^6^/mL in mice that are not treated with TG (RE M*ϕ*s). There were no differences in M*ϕ* number among groups treated with MTX and/or CsA and/or vehicles.

All animals were killed under halothane and exsanguinated immediately before these procedures.

### 2.4. Preparation of the soluble (S) and solubilized membrane-bound (M) fractions

M*ϕ* suspensions in 10 mM Tris-HCl buffer, pH 7.4 were sonicated at room temperature at amplitude 40 for 10 seconds. Sonicated M*ϕ*s were then ultracentrifuged (Hitachi model HIMAC CP60E) at 165000 X *g* for 35 minutes. The resulting supernatants were used to measure the S enzyme activities and protein concentrations. To avoid contamination with the S, the resulting pellet was washed three times with 10 mM Tris-HCl buffer, pH 7.4. The pellet was then homogenized for 30 seconds at 800 rpm (Contrac pestle mixer, FANEN, Brazil) in 10 mM Tris-HCl buffer, pH 7.4, plus 0.1% Triton X-100 and ultracentrifuged at 165000 X *g* for 35 minutes. The supernatants obtained were used to determine the M enzyme activities and protein concentrations. All steps were carried out at 4°C.

As a marker for the fractionation procedure, LDH activity was determined spectrophotometrically at 340 nm [25] in the S and M fractions of M*ϕ*s from all treated and control groups. Briefly, samples of 30 uL of S and M from M*ϕ*s were incubated with 270 uL of 100 mM phosphate buffer, pH 7.4, containing 200 mM NaCl and 1.6 mM sodium pyruvate solution plus 0.2 mM nicotinamide adenine dinucleotide, reduced form (NADH) disodium salt. Values of LDH activity were obtained by the results of subtraction of the absorbance at 340 nm read at 10 minutes from that read at 0 time of incubation at 37°C, and extrapolated by comparison with a standard curve of NADH. Student’s *t*-test was performed to compare the results of LDH between S and M fractions. The levels of LDH activity were similar to those previously reported [2], being higher in S than in M fractions (data not shown), which confirmed the efficiency of the adopted fractionation procedure. Moreover, these levels were not altered by MTX and/or CsA and/or vehicles treatments (data not shown).

### 2.5. Protein

Protein concentrations were measured in 40-uL samples at 630 nm by Bradford [26] method using Bio-Rad protein assay kit. Absorbance was read using the Bio-Tek Power Wave X^®^ spectrophotometer. Protein contents were extrapolated by comparison with respective standard curves of bovine serum albumin (BSA).

### 2.6. Peptidase assays

Peptidase activities were quantified on the basis of the amount of 4-methoxy-*β*-naphthylamine (for DPPIV) or *β*-naphthylamine (for POP) released as a result of the enzymatic activity of undiluted 50-uL samples of the S or M fractions from M*ϕ*s incubated at 37°C for 30 minutes in 96-well flat botton microplates (Corning Inc., USA) with 250 uL of each respective prewarmed substrate solution diluted to 0.2 mM (DPPIV) or 0.125 mM (POP) in corresponding 0.05 M buffers containing BSA 0.1 mg/mL. *β*-Naphthylamine or 4-methoxy-*β*-naphthylamine were estimated fluorometrically using the Bio-Tek FL600FA Microplate Fluorescence/Absorbance Reader, at 460/40 nm emission wavelength and 360/40 nm excitation wavelength in triplicate samples. The value of incubates at zero time (blank) was subtracted and the relative fluorescence was converted to picomoles of 4-methoxy-*β*-naphthylamine or *β*-naphthylamine by comparison with a correspondent standard curve. Peptidase activity was expressed as picomoles of substrate hydrolyzed per minute (UP) per milligram of protein. Assays were linear with respect to time of hydrolysis and protein content. DPPIV activity was measured by the method of Liu and Hansen [27] using H-Gly-Pro-4-methoxy-*β*-naphthylamide in Tris-HCl buffer, pH 8.3. POP activity was measured by the method of Zolfaghari et al. [28] using Z-Gly-Pro-*β*-naphthylamide in phosphate buffer, pH 7.4, with 2 mM dithiothreitol (DTT), without or with different concentrations of Z-Pro-Pro-OH (Z-pro-prolinal).

### 2.7. Materials

Commercially available cyclosporine A (Sandimmun^®^, Novartis, Brazil), methotrexate (Metrexato^®^, Blaüsiegel, Brazil), ricine oil (Sidepal, Brazil), Bio-Rad protein assay kit (Hercules, USA), Gly-Pro-4-methoxy-*β*-naphthylamide (Peninsula, USA), Z-Gly-Pro-*β*-naphthylamide, Z-Pro-Pro-OH (Bachem, USA) and Hema^3^ (Fisher Sci, USA). Bovine serum albumin, *β*-naphthylamine, gentian violet (crystal violet), halothane, 4-methoxy-*β*-naphthylamine, dithiothreitol, nicotinamide adenine dinucleotide, reduced form, disodium salt and sodium pyruvate were from Sigma, USA. All other reagents of analytical grade were from Merck, Brazil.

### 2.8. Statistical analysis

The data were analyzed statistically using GraphPad Prism^®^ and Instat^®^ softwares. Regression analyses were performed to obtain standard curves. Analysis of variance, ANOVA, was performed to compare values of the same enzyme activity from S or M among control groups, and to compare the values of POP activity under different concentrations of Z-pro-prolinal inhibitor. It was followed by student-newman-keuls test (SNK) when differences were detected. Student’s *t*-test was performed to compare the values of the same parameters between RE and TGE M*ϕ*s in each control group or between control and MTX- and/or CsA-treated animals on day 21, and to compare the values of latency of response-induced algesia between control and MTX- and/or CsA-treated animals along the treatments. Differences were considered statistically significant at a minimum level of *P* < .05.

## 3. RESULTS

Table 1 shows the peptidase activity levels of S and M fractions from RE and TGE M*ϕ*s of different controls. Saline per oral (p.o.) produced 1.5 to 2.5-fold reduction in soluble DPPIV and POP activities of TGE M*ϕ*s when compared to ricine oil s.c. or to ricine oil s.c. plus saline (p.o.). Membrane-bound DPPIV activity in TGE M*ϕ*s was also 4.8-fold lower after saline p.o. than after ricine oil plus saline treatment, which in this turn was 3.2-fold higher than after ricine oil s.c. administered alone. In RE M*ϕ*s, activity levels of both soluble DPPIV and POP obtained after all schemes of vehicle administration did not vary significantly, while membrane-bound DPPIV was about 5-fold higher after ricine oil plus saline treatment than ricine oil or saline administered alone. Comparisons between RE and TGE M*ϕ*s in each regimen of vehicle administration also revealed that soluble DPPIV activity of TGE M*ϕ*s was about 2-fold lower than RE M*ϕ*s from animals that received saline p.o. treatment. Membrane-bound DPPIV activity of TGE M*ϕ*s was 1.7-fold lower than RE M*ϕ*s from animals that received treatment with combined vehicles. Ricine oil s.c. or saline p.o administered alone produced a drop between 1.3 to 2.1 on POP activity levels of TGE M*ϕ*s compared to RE M*ϕ*s.

**Figure 1:**
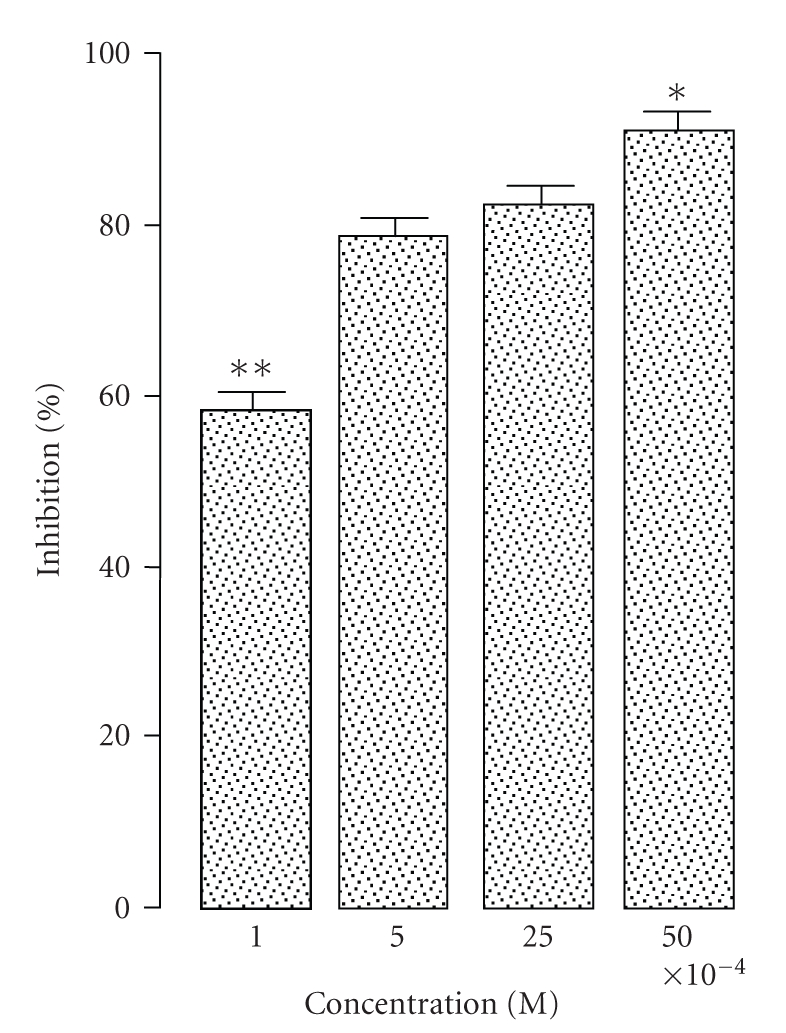
Effect of Z-pro-prolinal on soluble prolyl oligopeptidase activity of thioglycollate-elicited murine macrophages. The values (means ± SEM from 5 animals) were recorded in triplicate as the percentage of inhibition relative to control reactions (enzyme activity = 100%, percentage of inhibition = 0) which run simultaneously in absence of Z-pro-prolinal. All concentrations of Z-pro-prolinal produced lower levels of enzyme activity when compared to the control (unpaired two-sided Student's *t*-test, *P* < .05). * *P* < .05 in comparison to 5 × 10^−4^ M or × 10^−4^ M ; ** *P* < .001 in comparison to all other concentrations of Z-pro-prolinal (analysis of variance, ANOVA, followed by SNK test).

DPPIV activity of M*ϕ*s was previously demonstrated to be inhibited by diprotin A, a classical inhibitor of the canonical DPPIV [2]. Since POP activity of M*ϕ*s was surprisingly inhibited by classical aminopeptidase inhibitors [2], its susceptibility to a classical POP inhibitor, Z-pro-prolinal, was checked in the present study. As shown in Figure 1, POP activity of M*ϕ*s was inhibited (58–91%) by Z-pro-prolinal at the employed concentrations (1 to 50 × 10^−4^M).

**Figure 2:**
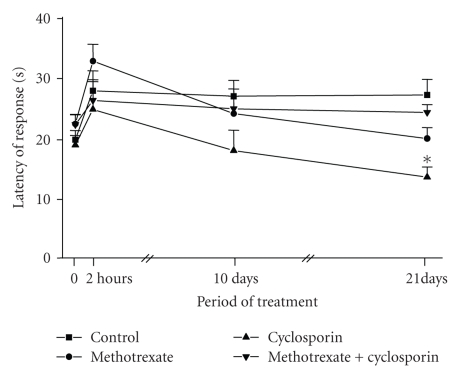
Latency of response-induced algesia of mice treated with methotrexate (MTX) and/or cyclosporine (CsA), or with ricine oil plus saline (control). Values are means ± SEM from 10–14 animals. * *P* < .001 in comparison to control (unpaired two-sided Student's *t*-test).

As shown in Figure 2, administration of vehicles, or MTX, or MTX plus CsA produced a similar reaction time to that of the thermal stimulus. On day 21, the administration of CsA alone decreased 0.58-fold of the reaction time when compared to the values obtained after the treatment with the combined vehicles.

**Figure 3:**
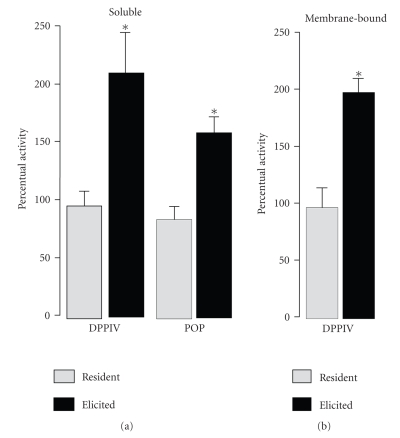
Percentual activity of soluble and membrane-bound dipeptidyl peptidase IV (DPPIV) and prolyl oligopeptidase (POP) activities in resident and thioglycollate-elicited murine macrophages from methotrexate-treated in relation to their respective controls (100%). Values are means ± SEM from 5 animals (assays made in triplicate). * *P* < .001 in comparison to control (unpaired two-sided Student's *t*-test).

Figure 3 shows that MTX treatment resulted in a significant rise of soluble activity levels of DPPIV (2.1-fold) and POP (1.6-fold) in the TGE M*ϕ*s when compared to those observed after the treatment with the respective vehicle. MTX treatment also resulted in a significant rise in membrane-bound DPPIV activity levels (2-fold) in TGE M*ϕ*s when compared to those observed after treatment with its respective vehicle (see Figure 3).

**Figure 4:**
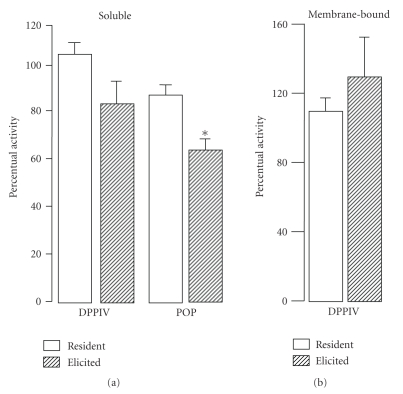
Percentual activity of soluble and membrane-bound dipeptidyl peptidase IV (DPPIV) and prolyl oligopeptidase (POP) activities in resident and thioglycollate-elicited murine macrophages from cyclosporine-treated in relation to the respective control (100%). Values are means ± SEM from 5 animals (assays made in triplicate). * *P* < .05 in comparison to control (unpaired two-sided Student's *t*-test).

Figure 4 shows that CsA treatment resulted in a drop of POP activity levels (0.79-fold) in the TGE M*ϕ*s when compared to those observed after the treatment with the respective vehicle. However, CsA treatment did not change the activity levels of soluble or membrane-bound DPPIV when compared to those observed after the treatment with the respective vehicle (see Figure 4).

**Figure 5:**
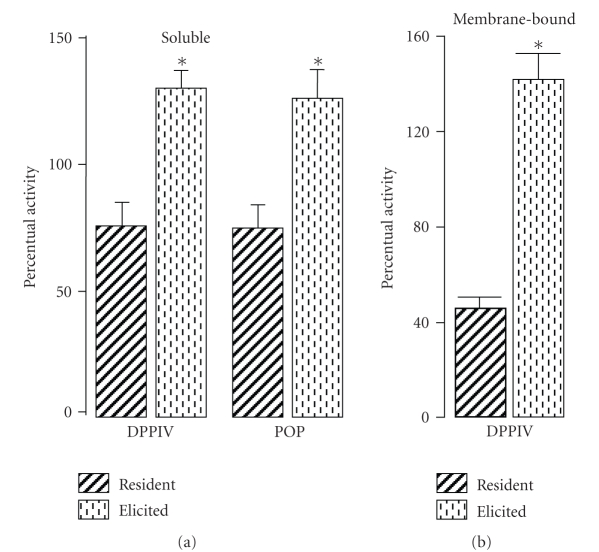
Percentual activity of soluble and membrane-bound dipeptidyl peptidase IV (DPPIV) and prolyl oligopeptidase (POP) activities in resident and thioglycollate-elicited murine macrophages from methotrexate plus cyclosporine-treated in relation to the respective control (100%). Values are means ± SEM from 5 animals (assays made in triplicate). * *P* < .001 in comparison to control (unpaired two-sided Student's *t*-test).

As shown in Figure 5, combined treatment with MTX and CsA promoted an increase in activity levels of soluble (1.5-fold) and membrane-bound (1.8-fold) DPPIV and soluble POP (1.8-fold) in the TGE M*ϕ*s when compared to those observed after treatment with the combined vehicles.

Figure 4 shows that CsA treatment resulted in a drop of POP activity levels (0.79-fold) in the TGE M*ϕ*s when compared to those observed after the treatment with the respective vehicle. However, CsA treatment did not change the activity levels of soluble or membrane-bound DPPIV when compared to those observed after the treatment with the respective vehicle (see Figure 4).

## 4. DISCUSSION

The intraperitoneal injection of TG increased about four times the obtained M*ϕ* number from all treatment groups (MTX and/or CsA and/or vehicles), after 4 days. However, the expression of enzyme activity adopted in the present study might not be correlated to cell number, since it was normalized by mg of protein in the cell homogenates, that is, enzyme activity = picomoles substrate hydrolyzed per mg of protein. As expected we detected stress-induced effects of adopted administration regimens on the activity levels of examined M*ϕ* peptidases. It is well-known that in response to certain physical stressors the release of neuropeptides from sensory nerves is increased, mainly substance P (or other inflammatory mediators), and in this turn, these neuropeptides promote the activation of M*ϕ*s [29]. It is noteworthy that DPPIV and POP presented higher activity levels in S and/or M fractions of RE and TGE M*ϕ*s from mice submitted to chronic s.c. (ricine oil treatment) and/or p.o. (saline treatment) administrations than those obtained without these stress stimuli [2]. However, in relation to s.c. regimen or to RE M*ϕ*s, inducible stress by p.o. regimen reduced the increment of peptidase activities in TGE M*ϕ*s. Overall, apart from these variations among different controls adopted in the present study, we elucidate that the regimen of MTX and/or CsA treatments differentially affected DPPIV and POP activities of murine M*ϕ*s, an effect that only occurred under elicited (or proinflammatory) status of these cells. The effect of MTX on DPPIV activity of proinflammatory TGE M*ϕ*s but not on RE M*ϕ*s suggested that DPPIV activity is relevant to the immunosuppressor/anti-inflammatory actions of MTX. MTX and CsA had opposite effects on POP activity of TGE M*ϕ*s, suggesting a drop of hydrolytic activity of TGE M*ϕ* POP on pain mediators. Since TGE M*ϕ*s are a model of proinflammatory M*ϕ*s and these cells are abundant in inflammatory processes, and the nervous system influences the peripheral inflammatory process, it is conceivable that the reduction of POP activity participates in the development of algesic hyperexcitability in CsA therapy. However, this algesic hyperexcitability could be attributed to an effect on the nervous system through which CsA treatment exacerbates the reaction to this stimulus. In this case, the altered reaction to acute thermal algesic stimulus might have an indirect participation of M*ϕ*s.

Changes on the activity levels of DPPIV-like enzyme(s) in TGE M*ϕ*s due to MTX treatment were particularly relevant, as these might participate within the pharmacological action of MTX through an increased ability of this cell to inactivate several susceptible substrates known to be inflammatory and/or immunological mediators. However, since MTX increased DPPIV activity in TGE M*ϕ*s but did not modify the reaction to the algesic stimulus, it is likely that this reaction is not related to an increased hydrolysis of substance P by this enzyme. Alternatively, only the reduction of DPPIV activity below a critical level, as observed here for POP activity of TGE M*ϕ*s from mice treated with CsA, could be related to hyperalgesia. DPPIV-like enzyme(s) exert different functions regarding cell type and intra- or extracellular conditions in which they are expressed [30], and they have been recognized as multifunctional proteins similar to the lymphocyte surface glycoprotein CD26 (EC 3.4.14.5). DPPIV activity was detected in M*ϕ*s [2] and only recently the subcellular fractionation of other leukocyte types has localized the preponderance of DPP8/9 protein to the cytosol and canonical EC 3.4.14.5 in the membrane fractions [31]. Based on that study, we can speculate that DPPIV activity in the soluble fraction of murine M*ϕ*s seems improbable to be EC 3.4.14.5, but most likely DPP8/9, although the classical DPPIV inhibitor diprotin A was effective to decrease the DPPIV activity in both S and M fractions of these cells [2]. Furthermore, since these activities have not been purified from murine M*ϕ*s, it remains to be investigated whether the changes in the DPPIV activity observed in the present study are accompanied by corresponding changes in the respective protein or mRNA expression. In general, these proline-specific dipeptidyl peptidases are unique among the aminopeptidases because of their superimposed ability to liberate Xaa-Pro and less efficiently Xaa-Ala dipeptides from the N-terminus of regulatory peptides. DPPIV-like enzymes act on peptide degradation (e.g., peptide hormone, various cytokines and growth factors), amino acid scavenging, cell-to-cell communication, signal transduction and adhesion, and as a receptor as well [30]. Recently, we have reported that CsA has no effect on basic and neutral aminopeptidase activities of TGE M*ϕ*s [32]. Here we evidenced that CsA has also no effect on DPPIV activity, but produced a reduction on POP activity levels in the proinflammatory model of TGE M*ϕ*s. POP is known as postproline cleaving enzyme activity, TRH-deaminase activity, or kininase B activity. The link between POP enzyme and inflammatory or autoimmune syndromes has been evidenced in some studies. In a mouse model of systemic lupus erythematosus, POP activity is increased in the spleen of diseased subjects when compared to controls. This increase is progressive with age and indicates that POP plays an important role in the immunopathological disturbances associated with this syndrome [33, 34]. Other links between POP and immunological disturbances were made when increased POP levels in synovial membrane preparations from patients suffering from rheumatoid arthritis were observed and it is also suggested that POP may play a significant role in the onset of osteoarthrosis [35, 36]. POP cleaves Pro-Xaa bonds in peptides that consist of an acyl-Yaa-Pro-Xaa sequence as found in nociceptive mediators such as bradykinin and substance P [37, 38], which can also be considered a link between inflammation and pain. It has been reported that aberrant pain perception and depressive symptoms in fibromyalgia are related to lower serum POP activity [39]. On the other hand, literature data about effects of CsA on nociception are controversial. Acute administration of CsA has been reported to reduce corticotropin-releasing factor-induced peripheral antinociception through effects on opioid-containing immune cells [40]. Headache symptoms in patients receiving CsA for organ transplantation have been connected with an endothelial dysfunction related to increased production of nitric oxide [41]. CsA has also been implicated in severe leg pain in patients with psoriatic arthritis [42]. Conversely, acute administration of CsA has been reported to induce an antinociceptive effect that involves the L-arginine-oxid nitric pathway, which is not mediated by opioid receptors [43], and also reduced inflammatory joint hyperalgesia in rat adjuvant-induced arthritis [44]. Moreover, it was reported that CsA can induce antinociception and increased plasma levels of met-enkephalin [45]. A possible explanation for these contraditory findings is that analgesic or algesic actions and their intensities vary according to prevalent cell types and mechanisms related to different pain stimuli and/or therapy regimen with CsA. Processes of sensitization (spontaneous pain and augmented pain response on noxious stimulation, and pain on normally nonpainful stimulation) are common in chronic peripheral inflammatory process such as arthritis, which has been currently treated with MTX and/or CsA [46–49]. This sensitization seems to involve bradykinin binding to nerve fibers receptors, while the expression of these receptors is upregulated during inflammation. In this turn, substance P promotes subsequent central sensitization to that produced by glutamate on spinal cord neurons [50]. However, at nerve fiber receptor level, the control of these mediators by enzymatic cleavage has not been investigated. Taken together, literature data, the effectiveness of immunosuppressive response to CsA on day 21 [23], and our data showing concomitant reduction of TGE M*ϕ* POP activity and tolerance to thermally-induced algesia by CsA, suggest a relationship between the observed effects of CsA on these proinflammatory cells and on the availability of bradykinin and/or substance P at receptor level of nerve fibers. Since MTX or MTX plus CsA increased POP activity but did not change the reaction to the same algesic stimulus, it is likely that besides POP activity other pain mediators are altered by the adopted treatment with CsA. Alternatively, only the reduction of POP activity below a critical level, as demonstrated here in the proinflammatory model of murine M*ϕ*s, could be related to hyperalgesia. It is also conceivable that murine M*ϕ* POP can potentially be a novel POP enzyme, since POP activity in murine M*ϕ*s was inhibited by classical aminopeptidase inhibitors [2] and, as demonstrated in the present study, by the classical POP inhibitor Z-pro-prolinal, at well-known effective concentrations [51]. Furthermore, residual Z-pro-prolinal-resistant POP activity in M*ϕ*s and in bovine serum could be similar [51]. To clarify these points, the identification of M*ϕ* POP protein and the effects on algesia using a selective inhibitor of M*ϕ* POP activity need further investigations.

In conclusion, our data indicate the participation of two representative prolyl peptidases in murine M*ϕ*s within the effects of MTX and/or CsA treatment, and provide scope for additional studies on the mechanism of action and efficacy of individual or combined therapy with both drugs in painful and inflammatory diseases.

## Figures and Tables

**Table 1:** Dipeptidyl peptidase IV (DPPIV) and prolyl oligopeptidase (POP) activities in soluble (S) and membrane-bound (M) fractions of resident (RE) and thioglycollate-elicited (TGE) macrophages from vehicle-treated animals (ricine oil s.c.= controls of cyclosporine; saline p.o.= controls of methotrexate; ricine oil s.c. plus saline p.o.= controls of methotrexate plus cyclosporine).

		Activity (UP/mg protein)			
Vehicle	Enzyme	S		M	
		RE	TGE	RE	TGE
Ricine oil	DPPIV	334 ± 42	359 ± 53*	112 ± 7	112 ± 26
Saline		391 ± 67	174 ± 35^a^	145 ± 43	73 ± 12
Ricine oil + saline		319 ± 23	357 ± 13**	605 ± 50***	355 ± 53***^b^

Ricine oil	POP	278 ± 16	200 ± 10**^c^	absent	
Saline		286 ± 44	133 ± 8 ^b^	absent	
Ricine oil + saline		320 ± 23	343 ± 18***	absent	

UP= picomoles substrate hydrolyzed per minute. Values are means ± SEM from 5 animals (assays made in triplicate). Comparisons among vehicle treatments regarding the same enzyme activity in each fraction and macrophage status (analysis of variance, ANOVA, followed by SNK test): * P < .05, ** P < .01 versus saline; ***P < .001 versus saline or ricine oil. Comparisons between TGE versus RE related to the same enzyme activity in each fraction and vehicle treatment (unpaired two-sided Student’s *t*-test): ^a^P < .03, *b*P < .01, ^c^P < .005.

## ABBREVIATIONS

BSA: Bovine serum albumin
CsA: Cyclosporine A
DPPIV: Dipeptidyl peptidase IV
DTT: Dithiothreitol
IFN gamma: Interferon-gamma
IL: Interleukin
LDH: Lactate dehydrogenase
M: Membrane-bound
MHC: Major histocompatibility complex
M*ϕ*: Macrophage
MTX: Methotrexate
NADH: Nicotinamide adenine dinucleotide reduced form
POP: Prolyl oligopeptidase
RE: Resident
S: Soluble
SNK: Student-Newman-Keuls test
TG: Thioglycollate broth medium
TGE: Thioglycollate broth medium-elicited
TNF alpha: Tumor necrosis factor-alpha
UP: Picomoles of substrate hydrolyzed per minute
